# Strategies for Implementing Occupational eMental Health Interventions: Scoping Review

**DOI:** 10.2196/34479

**Published:** 2022-06-01

**Authors:** Renaldo M Bernard, Claudia Toppo, Alberto Raggi, Marleen de Mul, Carlota de Miquel, Maria Teresa Pugliese, Christina M van der Feltz-Cornelis, Ana Ortiz-Tallo, Luis Salvador-Carulla, Sue Lukersmith, Leona Hakkaart-van Roijen, Dorota Merecz-Kot, Kaja Staszewska, Carla Sabariego

**Affiliations:** 1 Swiss Paraplegic Research Nottwil Switzerland; 2 Neurology, Public Health and Disability Unit Fondazione IRCCS Istituto Neurologico Carlo Besta Milano Italy; 3 Erasmus School of Health Policy & Management Erasmus University Rotterdam Netherlands; 4 Research, Innovation and Teaching Unit Parc Sanitari Sant Joan de Déu Universitat de Barcelona Sant Boi de Llobregat Spain; 5 Instituto de Salud Carlos III Centro de Investigación Biomédica en Red de Salud Mental Madrid Spain; 6 Department of Health Sciences Hull York Medical School University of York York United Kingdom; 7 Department of Psychiatry School of Medicine Universidad Autónoma de Madrid Madrid Spain; 8 Health Research Institute Faculty of Health University of Canberra Canberra Australia; 9 Institute of Psychology University of Lodz Lodz Poland; 10 Department of Health and Work Psychology Nofer Institute of Occupational Medicine Lodz Poland; 11 Department of Health Sciences and Medicine University of Lucerne Lucerne Switzerland; 12 Center for Rehabilitation in Global Health Systems World Health Organization Collaborating Center University of Lucerne Lucerne Switzerland

**Keywords:** implementation, mobile health, mHealth, mental health, eMental health, occupational health, barriers, facilitators, scoping review, mobile phone

## Abstract

**Background:**

The implementation of eMental health interventions, especially in the workplace, is a complex process. Therefore, learning from existing implementation strategies is imperative to ensure improvements in the adoption, development, and scalability of occupational eMental health (OeMH) interventions. However, the implementation strategies used for these interventions are often undocumented or inadequately reported and have not been systematically gathered across implementations in a way that can serve as a much-needed guide for researchers.

**Objective:**

The objective of this scoping review was to identify implementation strategies relevant to the uptake of OeMH interventions that target employees and detail the associated barriers and facilitation measures.

**Methods:**

A scoping review was conducted. The descriptive synthesis was guided by the RE-AIM (reach, effectiveness, adoption, implementation, and maintenance) framework and the Consolidated Framework for Implementation Research.

**Results:**

A total of 31 of 32,916 (0.09%) publications reporting the use of the web-, smartphone-, telephone-, and email-based OeMH interventions were included. In all, 98 implementation strategies, 114 barriers, and 131 facilitators were identified. The synthesis of barriers and facilitators produced 19 facilitation measures that provide initial recommendations for improving the implementation of OeMH interventions.

**Conclusions:**

This scoping review represents one of the first steps in a research agenda aimed at improving the implementation of OeMH interventions by systematically selecting, shaping, evaluating, and reporting implementation strategies. There is a dire need for improved reporting of implementation strategies and combining common implementation frameworks with more technology-centric implementation frameworks to fully capture the complexities of eHealth implementation. Future research should investigate a wider range of common implementation outcomes for OeMH interventions that also focus on a wider set of common mental health problems in the workplace. This scoping review’s findings can be critically leveraged by discerning decision-makers to improve the reach, effectiveness, adoption, implementation, and maintenance of OeMH interventions.

## Introduction

### Background

Mental health problems experienced by the working population are a global public health issue. Worldwide, more than 210 million people, representing 70% of those affected by common mental health disorders (eg, anxiety and mood disorders) are employed [1]. Several risk factors, including working conditions, workplace culture, and the nature of work, have been linked to occupational mental health [2-4]. Public health emergencies, such as the COVID-19 pandemic, are linked to specific stressors, including the threat of infection, social distancing measures, stigma, and job insecurity, which considerably increase the prevalence of mental health problems in the working population [5].

Occupational eMental health (OeMH) interventions significantly improve mental health in work settings [6]. OeMH interventions use information and communication technology, including internet- and web-based services, mobile apps, and wearable technologies, to deliver knowledge and services such as psychoeducation, workplace health promotion, psychological and medical treatment, and return to work assistance to employees [7,8]. OeMH interventions have the potential to be more available, accessible, and scalable than traditional interventions [9,10], especially in public health emergencies, leading to physical-distancing policies to contain the spread of threatening conditions such as COVID-19.

However, implementing OeMH interventions is a complex process characterized by unique challenges involving adherence to new and crude regulatory frameworks, interoperability and compatibility with existing systems and procedures, threats to employees, organizational privacy and security, and associated costs [11]. Newly introduced working arrangements in response to public health emergencies, such as the COVID-19 pandemic, could also compound existing implementation challenges and persist after the pandemic ends. Carefully developing and planning implementation strategies, which can be defined as a method or technique used to enhance the adoption, execution plan, and sustainability of an intervention [12], is therefore essential to guarantee the sustainable uptake of OeMH interventions by employers and employees.

Nonetheless, it is difficult to establish a best practice for the implementation of OeMH interventions. Implementation strategies are often inadequately documented and seldom evaluated and published [12,13], especially in comparison with studies on the effectiveness of interventions. Even when reported, implementation strategies have been discussed within a general context, and researchers have called for more tailored implementation strategies that focus on specific contexts [14], for instance health care [15]. Context encompasses the environment, broad setting, and circumstances (eg, systems and structures) in which an intervention is implemented and its associated characteristics [16]. It is a key component of several widely adopted implementation frameworks, as evident in the Consolidated Framework for Implementation Research (CFIR) [17]. Currently, those implementing new OeMH interventions are likely insufficiently informed about the procedure, strengths, and weaknesses of poorly documented implementation strategies, or uninformed about many potentially useful facilitators in this context. Furthermore, replicating positive results from similar implementations or overcoming barriers encountered in similar contexts would be challenging to achieve [18,19].

### Objectives

Therefore, a compilation of possible implementation strategies for OeMH interventions is critical to fostering improvements in their uptake and can serve as a reference for identifying and overcoming likely barriers and informing the future development of best practices. The objective of this scoping review was to identify implementation strategies relevant to the uptake of OeMH interventions that target employees and detail the associated barriers and facilitation measures. This scoping review would achieve these objectives by mapping the existing literature on the implementation of OeMH interventions and identifying gaps for future research. This work was conducted under the EMPOWER (European Platform to Promote Well-being and Health in the Workplace) project, funded by the European Commission, which investigates the impact of an eMental health platform aimed at preventing common mental health problems and reducing psychological distress in the workplace [20]. It is also one of the series of review papers on different aspects of the knowledge base related to the development of the EMPOWER platform.

## Methods

### Overview

A scoping review was conducted to identify implementation strategies relevant to the implementation of OeMH interventions and to describe related barriers and associated facilitation measures. The scoping review is an established method for assessing and mapping the extent of evidence to address and inform practice in a topic area [21-24]. The review proceeded through five stages as developed by Arksey and O’Malley [23], extended by Levac et al [22], and further modified by Westphaln et al [25] to accommodate a team-based approach: (1) identifying the research question; (2) identifying relevant studies; (3) selecting studies; (4) charting the data; and (5) collating, summarizing, and reporting the results. Accordingly, this scoping review provides an overview of the existing evidence without a formal assessment of the methodological quality. It is conducted and reported in accordance with the widely adopted PRISMA-ScR (Preferred Reporting Items for Systematic Reviews and Meta-Analyses extension for Scoping Reviews) [26] to help ensure a high level of methodological rigor and reporting quality.

### Search Strategy

Electronic bibliographic databases, including MEDLINE, Scopus, CINAHL Complete, PsycINFO, and Web of Science Core Collection, were searched to find eligible peer-reviewed and gray literature. Search terms were based on concepts related to mental health, digital tools, the workplace, and implementation strategies (Multimedia Appendix 1). The MEDLINE search strategy (Multimedia Appendix 1) was adapted for other databases using relevant syntax and keywords in consultation with all coauthors who are also experienced researchers in the area. Hand searching of the reference lists of included articles was also completed for further relevant literature not identified during the search of databases. Members of the EMPOWER Consortium (ie, mental health researchers, clinicians, and experts focusing on well-being in the workplace) were also requested to suggest potentially eligible references via email.

### Eligibility Criteria

Publications were eligible for inclusion if they described implementation strategies (ie, according to Proctor et al [12]) or related barriers or facilitation measures relevant to the uptake of OeMH interventions targeting employees. For example, all other eligibility criteria being met, approaches with the following characteristics would be considered: aim to introduce and encourage continued use of an intervention; prescribe actions in support of the intervention (eg, adaptations, fiscal strategies, and testing); and ensure that interventions can deliver intended benefits to the relevant organization over time, for instance, creating routine organizational policies or best practices. OeMH interventions are broadly defined here as mental health information and services delivered by information and communication technologies to employees [7,8]. This definition is consistent with the definition of eHealth [27,28], as well as the broader term digital health [27]. Studies with employed participants aged ≥18 years, that were written in English. and published between January 2010 and May 2021 were considered. Primary research studies, systematic reviews, books, and gray literature (eg, conference proceedings, theses, government documents, and professional publications) were considered. Gray literature, such as commentaries, letters to editors, and editorials, were excluded.

### Eligibility Assessment

A total of 10 researchers (AO-T, AR, CdM, CT, CMvdFC, DM-K, KS, MdM, MTP, and RMB), including psychologists, health scientists, and health economists, were involved in screening. To ensure consistency across researchers, they attended a web-based training workshop to practice the skills needed to reliably execute screening using the web and an app-based service Rayyan, Qatar Computing Research Institute [29]. A training set of 100 publications was screened by all workshop attendees. Screening decisions (ie, include, maybe, or exclude) were reviewed and discussed to clarify any misunderstandings and identify difficulties using Rayyan QCRI. Instructions not to use the natural language processing–, artificial intelligence (AI)-, and machine learning–based features offered in Rayyan QCRI as well as tips to overcome minor usability shortcomings were given. Screeners were randomly assigned a screening set, and a screener performed a second screening of 20% of titles, abstracts, and full texts, and 100% of the publications that received a *maybe* screening decision. All screenings were conducted independently to reduce the likelihood of reviewer bias [30] and inconsistencies in screening decisions were resolved in reconciliation meetings.

### Data Extraction and Synthesis of Results

In all, 5 researchers (AR, CdM, CT, MdM, and RMB), including psychologists and health scientists, of the 10 (50%) screeners, were involved in data extraction and attended a web-based training workshop focused on developing consistency across researchers by practicing the skills needed to reliably execute data extraction using a web-based data extraction form. The form was reviewed and improved for clarity regarding the questions asked, user friendliness, and efficiency of data entry. For instance, it was clarified that single-component implementation strategies were to be extracted, and any bundling of strategies (ie, multifaceted strategies) in publications to address a goal were to be noted. Each researcher was randomly assigned an equal number of included records, and a researcher reviewed the extracted data for all the included publications.

A descriptive synthesis was performed, where identified implementation strategies, barriers, and facilitators were collated and later summarized. The synthesis was conducted by 3 (CT, MdM, and RMB, ie, psychologists and health scientists) of the 6 (50%) researchers involved in data extraction and guided by the RE-AIM (reach, effectiveness, adoption, implementation, and maintenance) framework [31-33] and the CFIR [17] and further informed by the Expert Recommendations for Implementing Change [34]. RE-AIM and CFIR were chosen as they are widely used frameworks in implementation research (IR) [35] and were deemed by the authors to be the most comprehensive of the recently reviewed implementation frameworks [35] and most applicable to our objectives. The RE-AIM was originally developed as a framework for reporting findings regarding health promotion and disease management interventions in various settings. RE-AIM is used here to highlight essential strategy components with respect to its five steps: reach—the number of people who are willing to participate in a given initiative; effectiveness—the impact of an intervention on important outcomes (eg, individualistic and economic); adoption—the number of people or organizations who are willing to initiate and deliver an intervention; implementation—fidelity of delivery for the intervention including adaptations, costs, and consistency of delivery; and maintenance—sustained delivery and effects of an intervention after the associated initiative has ended. The CFIR unifies implementation theories to help build a robust implementation knowledge base across a wide range of studies, settings, contexts, and processes. The CFIR was used to provide a comprehensive view of multiple implementation contexts in which factors that might influence intervention implementation and effectiveness could be well detailed. Both frameworks determined the data for extraction: key publication characteristics, strategy definitions, key strategy implementation tasks, implementation processes, barriers and facilitators to strategy implementation, and any other data that holistically captured the complex and multilevel nature of strategy implementation were considered for data collection. Further synthesis of the identified barriers and facilitators produced recommendations for each relevant CFIR construct to improve the implementation of OeMH interventions.

## Results

**Figure 1:**
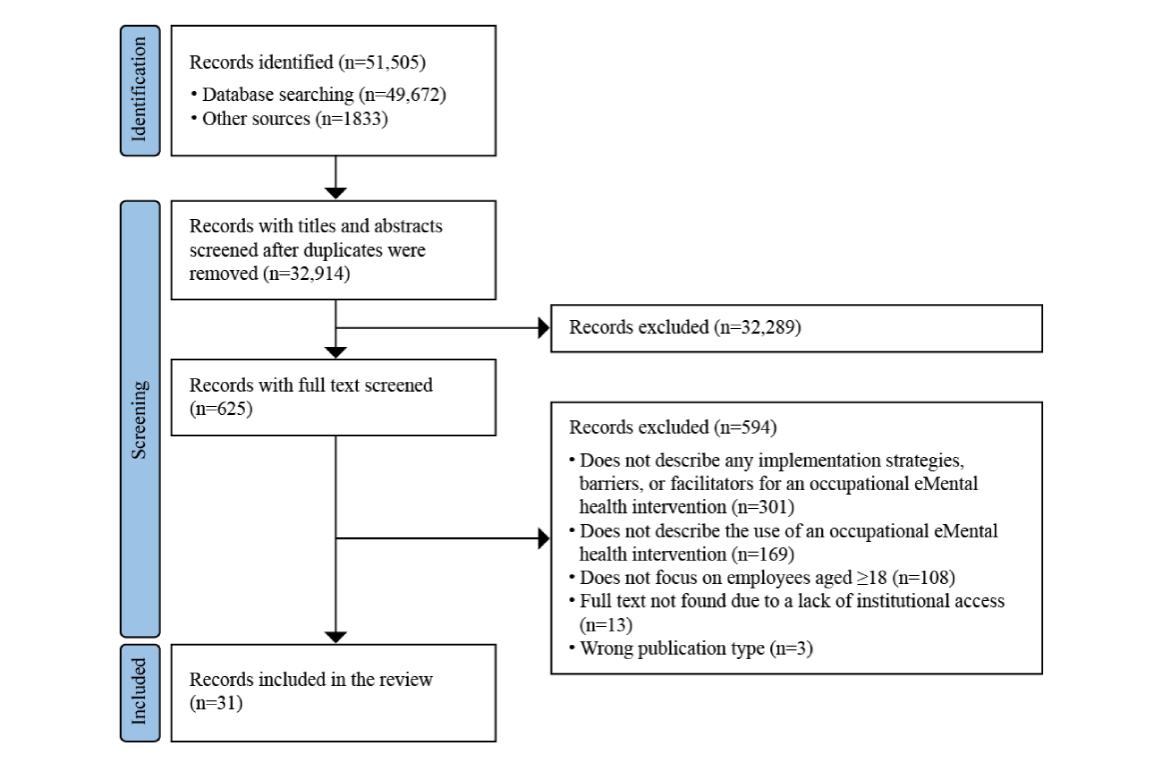
PRISMA (Preferred Reporting Items for Systematic Reviews and Meta-Analyses) flowchart of the review search, selection, and inclusion process.

A total of 31 publications were included in this scoping review (Table 1). Figure 1 details the methodological process followed, and a detailed itemization of the presented findings is provided in Multimedia Appendices 2 and 3.

**Table 1:** Characteristics of included publications and interventions.

Citation and year of publication	Study aim and methods (n)	Country of implementation, industry, and participating organizations (n)	Intervention name, aim, and target conditions	Digital technologies used
[36], 2020	To develop, implement, and evaluate the intervention; survey (503), interviews (19), and focus groups (32)	United Kingdom; human health and social work activities; 7	Healthier Outcomes at Work Social Work Project; improve and manage; workplace stress and mental well-being	Smartphone app
[37],^a^ 2020	To describe the intervention’s implementation; protocol—pilot randomized controlled trial (106)	China; human health and social work activities; 1	Step-by-Step F; improve; depressive symptoms and anxiety symptoms	Web-based and smartphone app
[38],^a^ 2020	To describe the evaluation of the intervention’s implementation; protocol—focus groups (N/R^b^)	Germany; agriculture, forestry, and fishing; N/A^c^	With us in balance; prevent; stress-related disorders, anxiety disorders, mood disorders, substance-related and addictive disorders, insomnia, and chronic pain	Web-based and telephone
[39], 2020	To examine perspectives on the role and legitimacy of the intervention; interviews (32) and focus group (14)	Sweden; N/R; N/A	mWorks; support; common mental disorders	Smartphone app
[40],^a^ 2020	To conduct preliminary evaluation of the intervention; pilot—usability study (81)	Australia; N/S^d^; N/R	Anchored app; assess, improve, and monitor; depression, workplace stress, and mental well-being	Smartphone app
[41],^a^ 2020	To rapidly develop and evaluate the intervention; stakeholder consultation groups (97), peer review panel (10), and intervention fidelity and implementation testing (55)	United Kingdom; human health and social work activities; N/R	Psychological Well-being in Healthcare Workers: Mitigating the Impacts of COVID-19; support and manage; workplace stress and mental well-being	Web-based
[42], 2019	To evaluate the feasibility, outcome, and acceptability of the intervention; proof-of-concept—survey (33)	United Kingdom; public administration and defense and compulsory social security; 2	Self-confidence webinar program; improve; mood disorders and depression	Web-based
[43], 2019	To evaluate engagement with the intervention; survey (149)	United States; public administration and defense and compulsory social security; 20	Stress Reduction Training for 9-1-1 Telecommunicators; improve and promote; workplace stress	Web-based
[44], 2019	To conduct formative evaluation of the intervention; interviews (24)	New Zealand; public administration and defense and compulsory social security; N/R	N/R; improve; stigma and discrimination	Web-based
[45], 2018	To evaluate adherence to the intervention; randomized controlled study (563)	Sweden; education; 21	N/R; improve and promote; workplace stress, occupational health, and sleep quality	Web-based
[46], 2018	To evaluate the helpfulness of the intervention; web-based survey (22) and focus groups (2)	United States; human health and social work activities; 1	Paving the Path to Mindfulness Website; improve; burnout and workplace stress	Web-based
[47], 2018	To evaluate acceptance and barriers to the uptake of OeMH^e^ interventions; survey (3294)	N/A; N/A; N/A	N/A; manage; work-related distress	N/S
[48],^a^ 2018	To evaluate the implementation strategy used; controlled trial (221)	The Netherlands; human health and social work activities; 1	Stress Prevention@Work; improve and prevent; workplace stress	Web-based
[49],^a^ 2018	To evaluate the effectiveness of the implementation strategy used; follow-up controlled trial (252)	The Netherlands; human health and social work activities; 1	Stress Prevention@Work (SP@W); assess, improve, and prevent; workplace stress	Web-based
[50],^a^ 2018	To identify key correlates of intention to use OeMH interventions; survey (1364)	China; human health and social work activities; N/A	N/A; N/A; mental health conditions	Web-based and smartphone app
[51],^a^ 2018	To evaluate use of OeMH; log data and survey (1284)	Sweden; N/R; 6	N/R; improve, monitor, promote, and support; workplace stress and mental well-being	Web-based
[52], 2018	To develop and pilot-test the usability, acceptability, feasibility, and preliminary effectiveness of the intervention; prototype testing (21) and effectiveness and feasibility pilot study (84)	Australia; agriculture, forestry and fishing, manufacturing, and logistics; 3	HeadGear; improve; depressive symptoms	Smartphone app
[53],^a^ 2018	To identify facilitators and barriers to engagement with OeMH interventions; interviews (18)	United Kingdom; information and communication, public administration and defense, education, and other service activities; 6	WorkGuru; improve; workplace stress	Web-based
[54],^a^ 2017	To conduct process evaluation of the intervention; survey, log data, interviews, and observations (132)	The Netherlands; N/R; 2	eHealth module embedded in collaborative occupational health care; improve and monitor; mental well-being and return to work	Web-based
[55], 2017	To compare engagement with(out) a discussion group; pilot—3-arm randomized controlled trial (84)	United Kingdom; information and communication, public administration and defense, compulsory social security, education, and third sector organization; 6	WorkGuru; educate, improve, and monitor; workplace stress and nonworkplace stress	Web-based
[56], 2016	To investigate the influence of guidance formats on adherence of the intervention; pooled data from randomized controlled trials (395)	Germany; N/R; N/R	GET.ON Stress; improve and manage; workplace stress	Smartphone app
[57], 2016	To investigate men’s preferences for OeMH interventions’ design features; cross-sectional survey (841)	Canada; N/A; N/A	N/A; N/A; workplace stress and major depression	N/A
[58], 2016	To describe the development, implementation, and outcomes of; survey (1333)	United States; human health and social work activities; 1	Sleep Smart; improve and promote; poor sleep health	Email
[8],^a^ 2016	To describe approaches to and perspectives on OeMH interventions; N/A (N/A)	N/A; N/S; N/A	N/A; N/A; N/A	N/S
[59], 2016	To evaluate the potential effectiveness of the intervention and the effect of an online facilitated discussion group on engagement; protocol—3-arm randomized controlled trial (90)	United Kingdom; N/R; N/A	WorkGuru; educate, improve, and monitor; Workplace stress and nonworkplace stress	Web-based
[60], 2015	To describe the development the intervention; individual (34) and focus group (18) feedback sessions	United States; public administration and defense, compulsory social security, and human health and social work activities; N/R	Coming Home and Moving Forward; improve; stress-related disorders and substance-related and addictive disorders	Web-based
[61], 2014	To investigate users’ views on two different technologies for an OeMH intervention; survey within randomized controlled trial (637)	United Kingdom; transportation and storage, information and communication, and human health and social work activities; 3	Mood GYM; improve; mood disorders	Web-based
[62],^a^ 2014	To contrast the role of differing managerial levels during the implementation of an OeMH; interviews (29)	Sweden; information and communication; public administration and defense; compulsory social security; education; and arts, entertainment, and recreation; 9	N/R; assess, improve, monitor, and promote; mental well-being	Web-based
[63],^a^ 2014	To assess the feasibility of the intervention and explore barriers and /facilitators for the implementation of the intervention; process evaluation alongside a randomized controlled trial (116)	The Netherlands; financial and insurance activities; professional, scientific, and technical activities; public administration and defense; compulsory social security; and education; 6	Happy Work; improve and prevent; depressive symptoms	Web-based
[64],^a^ 2013	To describe the development and implementation of the intervention; N/A (N/A)	International; N/R; N/R	HealthWatch; manage, prevent, and promote; mental well-being	Web-based
[65], 2010	To investigate determinants of high use of the intervention; randomized controlled intervention (303)	Sweden; information and communication and arts, entertainment, and recreation; N/R	N/R; assess, monitor, and promote; workplace stress	Web-based

^a^Focused on implementation.

^b^N/R: not reported.

^c^N/A: not applicable.

^d^N/S: not specified.

^e^OeMH: occupational eMental health.

### Publication Characteristics

The 31 included publications comprised 28 journal articles [36-45,47,48,50-57,59-63], 2 book chapters [8,64], and a doctoral dissertation [46] (Tables 1 and 2). Most (25/31, 81%) [8,36-47,49-59] of the included articles were published between 2015 and 2021 and were mainly primary studies (23/31, 74%) [36,39,41-51,53,54,56-58,60-63,65]. Of the 31 included publications on OeMH interventions, 14 (45%) focused on their implementation [8,37,38,40,41,49-51,53,54,62-64] and 17 (55%) did not focus on their implementation but had results or noted implications related to their implementation [36,39,42-47,52,55-61,65].

**Table 2:** Summary of study characteristics (N=31).

Characteristics and citations		Frequency, n (%)
**Publication type**		
	Book chapter [8,64]	2 (6)
	Doctoral dissertation [46]	1 (3)
	Journal article [36-45,47,48,50-57,59-63]	28 (90)
**Publication year**		
	2010 [65]	1 (3)
	2013 [64]	1 (3)
	2014 [61-63]	3 (10)
	2015 [60]	1 (3)
	2016 [8,56-59]	5 (16)
	2017 [54,55]	2 (6)
	2018 [45-53]	9 (29)
	2019 [42-44]	3 (10)
	2020 [36-41]	6 (19)
**Study type**		
	Narrative literature review [8,65]	2 (6)
	Pilot [40,52,55]	3 (10)
	Primary study [36,39,41-51,53,54,56-58,60-63,65]	23 (74)
	Protocol [37,38,59]	3 (10)

### Intervention Characteristics

A total of 24 interventions were reported in 27 studies [36-46,49,51-56,58-65] (Table 3). These interventions were largely web-based (n=16, 67%%) [41-46,48,49,51,53-55,60-65] and most aimed to improve (n=19, 79%) [36,37,40,42-46,49,51-56,58-63] and, to a lesser extent, educate users about mental health problems. Most interventions have focused on stress-related disorders and symptoms (n=17, 71%) [8,36-47,49-51,53-63,65], but a wide range of mental health problems (eg, burnout, anxiety disorders, and substance-related disorders) have also been covered to some extent. Where reported [36,38,41,43-46,49,51-53,55,56,58-65], these interventions (n=19, 79%) largely targeted employees in professional occupations (eg, teachers and physicians). Most of these interventions were made available in specific countries, mainly in Europe (n=15, 63%%) [36,38,39,41,42,45,49,51,53-56,59,61-65], except for one that was available internationally (n=1, 4%) [64]. Standardized information about these 24 interventions, including year of launch, language, number of employees and employers interested in and who adopted the app, organizational size, and internal policies, was not clearly reported where relevant and could not be accurately extracted in detail.

**Table 3:** Summary of intervention characteristics (N=24).

Characteristics and citations		Frequency, n (%)
**Technology**		
	Smartphone [36,39,40,52,56]	5 (21)
	Web [41-46,48,49,51,53-55,60-65]	16 (67)
	Web and smartphone [37]	1 (4)
	Web and telephone [38]	1 (4)
	Email [58]	1 (4)
**Aim**		
	Assess [40,49,62,65]	4 (17)
	Educate [55,59]	1 (4)
	Improve [36,37,40,42-46,49,51-56,58-63]	19 (79)
	Manage [36,41,47,56,64]	5 (21)
	Monitor [40,51,54,55,59,62,65]	6 (25)
	Prevent [38,49,63,64]	4 (17)
	Promote [43,45,51,58,62,64,65]	7 (29)
	Support [39,41,51]	3 (13)
**Target mental health problem**		
	Anxiety disorders and symptoms [37,38]	2 (8)
	Burnout [46]	1 (4)
	Chronic pain [38]	1 (4)
	Common mental disorders [39]	1 (4)
	Mood disorders and symptoms [37,38,40,42,52,57,61,63]	8 (33)
	Return to work [54]	1 (4)
	Sleep problems [38,45,58]	3 (13)
	Substance-related and addictive disorders [38,60]	2 (8)
	Stigma and discrimination [44]	1 (4)
	Stress-related disorders and symptoms [8,36-47,49-51,53-63,65]	17 (71)
	Well-being problems [36,40,41,51,54,62,64]	7 (29)
**Country of implementation**		
	Australia [40,52]	2 (8)
	Canada [43]	1 (4)
	China [37]	1 (4)
	Germany [38,56]	2 (8)
	International [64]	1 (4)
	The Netherlands [49,54,63]	3 (13)
	New Zealand [44]	1 (4)
	Sweden [39,45,51,62,65]	5 (21)
	United Kingdom [36,41,42,53,55,59,61]	5 (21)
	United States [43,46,58,60]	4 (17)
**Target occupational groups**		
	Armed forces occupations [44,60]	2 (8)
	Clerical support worker [45,55,61,62]	4 (17)
	Elementary occupations (eg, cleaners and laborers) [45]	1 (4)
	Managers (eg, chief executive officer) [41,55,61]	3 (13)
	Not reported [37,39,40,42,54]	5 (21)
	Plant and machine operators and assemblers [61]	1 (4)
	Professionals (eg, teachers and physicians) [41,43,45,46,49,55,58,61-63]	10 (42)
	Service and sales workers [45,61,65]	3 (13)
	Social workers (ie, specifically child and family social workers) [36]	1 (4)
	Skilled agricultural, forestry, and fishery workers [38,61]	2 (8)
	Technicians and associate professionals [36,41,55,61,62,65]	6 (25)

### Implementation Strategies

#### Overview

Overall, 98 examples of implementation strategies were identified (Multimedia Appendix 2). Table 4 categorizes these strategies into 17 discrete implementation strategies and maps them onto relevant RE-AIM domains based on the perceived intent of the themes. Each discrete implementation strategy is reported with the percentage and absolute number of defining strategy examples in relation to the 98 examples. Although evaluating effectiveness strategies is beyond the scope of this review, relevant effectiveness data could not be presented as they were largely absent or incomplete.

A total of 36 examples of implementation strategies were extracted from publications that focused on the implementation of OeMH interventions, and 62 from publications that reported results or noted implications related to their implementation. There were no notable differences other than the larger number of examples extracted from the latter group so strategy examples would not be reported separately. Most strategy examples were organized under implementation (61/98, 62%), followed by reach (27/98, 28%), effectiveness (19/98), adoption (17/98, 17%), and maintenance (8/98, 8%). A couple of strategy examples were organized into multiple domains (6/98, 6%). The following sections provide a descriptive summary of the strategy examples categorized in each RE-AIM domain.

**Table 4:** Discrete implementation strategies mapped to relevant RE-AIM (reach, effectiveness, adoption, implementation, and maintenance) domains (N=98).

Discrete implementation strategies—proportion of strategy examples; n (%)	Example strategy	Relevant RE-AIM domains
Develop and organize implementation quality monitoring systems and act on insights in a timely manner where feasible; 17 (17)	Improve maintenance and adherence through the timely presentation of findings from monthly user feedback surveys where after follow-up actions can be immediately applied to the intervention [64]	Effectiveness, implementation, and maintenance
Assess for readiness and tailor strategies to address identified barriers and benefit from facilitators; 13 (13)	Filled knowledge gaps surrounding the effectiveness of eMental health interventions in the workplace by conducting systematic reviews on relevant topics [52]	Reach, effectiveness, implementation, and maintenance
Use mass media to increase reach; 9 (9)	Users were recruited by sharing information about the intervention through advertisements distributed via email and the organizations’ intranet and magazine [59]	Reach
Capture local knowledge from implementation sites and involve users early in the implementation and intervention development effort; 8 (8)	A consultation process was carried out with users, clinical psychologists, psychiatrists, information technology professionals, and design and user experience specialists to ensure the app’s content and design appealed to a broad range of workers from different industries [40]	Reach, effectiveness, adoption, implementation, and maintenance
Promote adaptability in the intervention to meet local needs without compromising fidelity; 8 (8)	Interventions were improved and adapted to each participating organization based on user feedback [36]	Reach, effectiveness, adoption, implementation, and maintenance
Send reminders; 7 (7)	Automatic email reminders were sent based on user-determined intervals and user inactivity [45]	Implementation
Provide support to users during the intervention; 6 (6)	Users were able to contact the intervention coach at any time to ask for feedback, additional help, or advice and the coach would respond within 24 hours [59]	Implementation
Conduct educational meetings; 5 (5)	Senior and middle management–led introductory seminars with employees that aimed to explain the intervention, secure acceptance, provide answers to questions, and inspire their participation [64]	Reach, adoption, and implementation
Provide incentives; 5 (5)	Users received a certificate of completion and the training was recognized as continuing education toward the renewal of their professional certification [43]	Reach and implementation
Identify and prepare organizational champions who will dedicate themselves to supporting, marketing, and driving the implementation; 4 (4)	Identification of champions at the implementation site facilitated organizational and employee buy-in [46]	Reach, adoption, implementation, and maintenance
Involve senior management; 4 (4)	The program was developed as a quality improvement project by the hospital and all research procedures (ie, retrospectively reviewing these outcomes) were approved by the institutional review board at the hospital [58]	Reach, adoption, implementation, and maintenance
Provide opportunities for users to obtain feedback on progress; 4 (4)	Participants received immediate and automatic tailored feedback and could monitor their own responses and trends over time [45]	Implementation
Stage implementation scale-up; 4 (4)	Conducted a pilot study aimed at assessing the usability, feasibility, acceptability, and preliminary effects of an app-based intervention designed to target depressive symptoms in a stressed working population [55]	Effectiveness and implementation
Customize recruitment activities to enhance reach; 3 (3)	When recruitment efforts did not attract enough participants, executives with the largest workforces in the region and industry were contacted directly via telephone and offered enrollment [45]	Reach and adoption
Develop and distribute educational materials; 3 (3)	All participants who returned the consent form received an email welcoming them to the study and explaining how to log in and use their personal webpage for the stress management program [65]	Reach, adoption, and implementation
Provide immediate opportunities to demonstrate commitment; 3 (3)	Management representatives were offered spots to enroll their organizations immediately after educational meetings about the intervention or to enroll at a later time [45]	Reach and adoption
Use advisory boards and workgroups to provide input and advice on implementation and improvements; 3 (3)	Systematic feedback was sought from researchers, expert clinicians, and veterans on the program and its content [60]	Implementation

#### Reach

Mass media services were mainly used to increase reach (9/27, 33%). Examples include email [36,43,46,52,58,59,63], industry publications [43,59], targeted web-based advertisements (eg, Facebook) [40,52], and the organizations’ intranet [59,61,63]. The provision of attractive incentives for participation (5/27, 19%) included monetary remuneration [60], vouchers [40], points for employee reward schemes [58], educational credits for professional certifications [43], and additional medical benefits [65]. Other strategies included engaging potential users through educational meetings [62] and materials [65] (2/27, 7%), employees tasked with the responsibility of supporting the implementation of the intervention [46,61] (2/27, 7%), and well-timed opportunities to commit [64,65] (2/27, 7%). Local barriers to increasing reach among target users were identified through consultations with implementation sites, eligible users, and literature [52,61,64] (3/27, 11%), and recruitment activities were later modified to avoid or overcome these barriers where possible [63] (4/27, 15%).

#### Effectiveness

Strategies to improve the effectiveness of the intervention mainly relied on insights obtained from a diverse group of professionals in relevant fields, representatives of implementation sites, target users, and intervention use data (12/19, 63%). This insight was captured through stakeholder consultations [41], steering group interviews and focus groups with target users [36], peer review panels [41], and user experience research [36,37,41,52,53,63] throughout the implementation process. Several strategies adopted an incremental approach to implementation [52,55,59] (3/19, 16%), fostered adaptability in the intervention to adequately meet the local needs [60] (2/19, 11%), or implemented measures to avoid or mitigate identified barriers that could negatively impact the effectiveness of the particular intervention [49,52] (2/19, 11%).

#### Adoption

Sharing and discussing details about the proposed intervention with decision-makers was the most commonly used adoption strategy. This involved conducting educational meetings [45,62,64,65] (4/17, 24%) and distributing educational materials about the intervention [45] (1/17, 6%). Engaging senior management and others from the organization to identify necessary adaptations for intervention to succeed in the organization was also common. These strategies involved organizational stakeholders early in the intervention development process [49,64] (2/17, 12%) to adapt the intervention to meet special organizational needs without compromising fidelity [64] (1/17, 6%) and address other identified barriers [39] (1/17, 6%). Some strategies also identified staff members who could dedicate themselves to supporting, marketing, and driving the implementation within the organization, as this was expected to increase the likelihood of success [46,49] (2/17, 12%). The provision of immediate opportunities for decision-makers to confirm their commitment to adopt the intervention was also used [45] (1/17, 6%).

#### Implementation

Implementation strategies focused on adapting interventions and customizing the implementation process to implementation settings, monitoring the consistency of delivery, and providing various forms of support as needed. Implementers underwent training, subscribed to a common protocol, and had their work reviewed to help ensure fidelity. Some implementation strategies were continuously monitored using both qualitative and quantitative methods, including surveys, implementation reviews, process evaluations, and other similar methods [36-38,41,49,54,56,61,63,64] (16/61, 26%). A diverse group of stakeholders were involved in the assessments across the included studies. These assessments focused on measuring effectiveness, acceptability, and engagement [36-38,41,49,54,56,61,63,64] (16/61, 26%). Findings were regularly applied quickly to overcome identified barriers and improve ongoing implementation processes [36,60,63,64] (10/61, 16%). Some support options included a reminder feature (7/61, 11%) where users could set their own reminder notifications [46] and be notified when their participation level was too low [45,56,63] or when new updates became available [43,46].

#### Maintenance

Maintenance strategies involved changes at the organizational level, where accommodating work conditions [43,55] (2/8, 25%) and support staff [58] (1/8, 13%) were sometimes arranged. Embedding interventions within existing employee programs was also expected to help sustain the use of the intervention [58] (1/8, 13%). Special monitoring measures (eg, postintervention acceptability surveys and opportunities for monthly user feedback) were also established to provide insight into how benefits to users could be sustained after the initiative had officially ended [37,64] (2/8, 25%).

### Barriers and Facilitators

#### Overview

The included publications reported 114 barriers and 131 facilitation measures (Multimedia Appendix 3), and 28 barriers were accompanied by facilitation measures. There were no notable differences between barriers and facilitators extracted from publications that focused on the implementation of OeMH interventions (108/217, 49.8%) or that reported results or noted implications related to their implementation (109/217, 50.2%) so these will be reported together. Examples of barriers and facilitators organized by the relevant CFIR domains and associated constructs are provided in the corresponding tables. Most of the 217 identified barriers and facilitation measures were related to key attributes of interventions that influence successful implementation (103/217, 47.5%), followed by the inner setting of the organization (87/217, 40.1%), individual characteristics of target users (25/217, 11.5%), and the outer setting of the organization (2/217, 0.9%). The highest number of barriers were categorized under the inner setting (54/114, 47.4%), followed by intervention characteristics (35/114, 30.7%), individual characteristics of target users (22/114, 19.3%), and the outer setting of the organization (2/114, 1.8%) domains. The highest number of facilitators were categorized under intervention characteristics (77/131, 58.8%), followed by inner setting (44/131, 33.6%), individual characteristics of target users (9/131, 6.9%), and outer setting of the organization (1/131, 0.8%) domains.

#### Intervention Characteristics

Numerous barriers and facilitators were identified regarding how the interventions were bundled, presented, and assembled (ie, design quality and packaging) (Table 5). Participants from several studies considered web-based platforms to be an impersonal medium (eg, no face-to-face contact or human interaction) [53,57,61], and some saw its use as inappropriate for helping with sensitive topics such as mental health problems [44]. Several usability issues (eg, poor accessibility, technical issues, unclear navigational elements and user interface, and overly effortful tasks) have also emerged as barriers [40,52,53,60,61,64]. Accordingly, ensuring good usability [8,39,40,53,57,64] and considering individual factors (eg, high impulsivity benefits from continuous motivational components) [39,45,57,65] in the design were also often reported as facilitators.

The stakeholders’ perceptions of the evidence supporting the effectiveness of the proposed occupational mental interventions were influenced by several factors. The barriers included between-group contamination due to limited randomization at the individual level, unrepresentativeness of samples used for the general workforce, use of new or adapted measures with low reliability [55], and type 1 errors [58]. Identified facilitators focused on the including diverse samples (eg, including underrepresented industries and occupations) [55], collecting comparable demographic data [55], including comprehensive engagement measures [55], presenting interventions based on credible information highly relevant to target employees [57], using control conditions when evaluating effectiveness [42,58], providing evidence from similar interventions that demonstrate effectiveness [65], and conducting comprehensive and ongoing process evaluations to inform implementation [51,63].

**Table 5:** Examples of barriers and facilitators organized under the intervention characteristics Consolidated Framework for Implementation Research domain (N=217).

Relevant associated construct—proportion of barriers and facilitators; n (%) and brief description	Example of identified barriers	Example of identified facilitators
Evidence strength and quality; 15 (6.9); stakeholders’ perceptions of the quality and validity of evidence supporting the belief that the intervention will have desired outcomes	Using newly created or adapted measures demonstrating low reliability negatively impacts the strength of findings [55]	Providing evidence from other programs and interventions could be a strategy (oral presentations or reading materials) to demonstrate likely effectiveness [65]
Relative advantage; 2 (0.9); stakeholders’ perception of the advantage of implementing the intervention versus an alternative solution	Possible low motivation from employers and organization in their employees return to work as they came from small- to medium-sized companies that had insurance for the costs of sickness absence [54]	The lack of a previous existing intervention for well-being in the organization, except for the intranet, which was difficult to use, so the app resulted to be a huge advantage for employees [36]
Adaptability; 4 (1.8); the degree to which an intervention can be adapted, tailored, refined, or reinvented to meet local needs	Materials presented in a modular format that had to be completed start to finish in a single sitting or in a set order [61]	Possibility to use the program at their own pace [60]
Design quality and packaging; 80 (36.9); perceived excellence in how the intervention is bundled, presented, and assembled	Usability was affected by unclear navigational elements and user interface [40]	Improving usability based on participant and expert feedback [40]

#### Outer Setting

Strict external policies and failure of interventions to meet patient needs erected several barriers to the implementation of OeMH interventions (Table 6). For example, strict legislation and policies regarding privacy and confidentiality were highlighted as potential reasons for the reduced adoption of interventions based on innovative technologies [39]. Moreover, failure to maintain employees’ confidentiality during these programs was believed to discourage the use of interventions for fear of being vulnerable to privacy breaches by employers [59]. The sole facilitation measure identified for this CFIR domain also addresses this point by urging implementers to find ways to maintain employee confidentiality [59].

**Table 6:** Examples of barriers and facilitators organized under the outer setting Consolidated Framework for Implementation Research domain (N=217).

Relevant associated construct—proportion of barriers and facilitators; n (%) and brief description	Example of identified barriers	Example of identified facilitators
External policy and incentives; 1 (0.5); a broad construct that includes external strategies to spread interventions including policy and regulations (governmental or other central entity), external mandates, recommendations and guidelines, pay for performance, collaboratives, and public or benchmark reporting	The surrounding legislation and policy regulation of privacy and confidentiality may make it difficult to use innovative technology [39]	—^a^
Patient needs and resources; 1 (0.5); the extent to which patient needs, as well as barriers and facilitators to meet those needs, are accurately known and prioritized by the organization	Reluctancy of the potential participants in participating for fear of demonstrating vulnerability [45]	Maintaining confidentiality between employee and employer [45]

^a^No facilitator reported.

#### Inner Setting

Many publications have identified the lack of resources dedicated to implementation as a major barrier (Table 7). For example, there is a lack of time for employees to use the intervention [40,48,49,52,53,59,61], funds to meet additional costs [39], unreliable systems that lead to data loss [58], inflexible participation times [42], lack of workspaces to avoid office distractions and private spaces [53] when completing interventions [61], low technology (eg, computers and email) adoption by the organization [64], little support from the app or implementor [54], and insufficient resources for piloting [62]. Some interventions were also inadequately adjusted to organizational processes [36,49,54,63] and insufficiently tailored to the work situation and culture [42,48,54,58,63]. Organizational restructuring has also been identified as a barrier to successful implementation and should be considered during implementation planning [42,48,63].

Several facilitators have also been identified. For example, it was recommended for employers to arrange dedicated time for employees to participate in the intervention [59]; to allow employees flexibility regarding the time, place, and pace when completing the intervention [53]; to offer an option for employees to use the intervention in a private workspace [53]; to provide recordings of any live sessions with feedback options [42]; and to encourage employee access to or ownership of technology (eg, smartphone) in use [50]. Intervention creators can also support employers with recruitment [55], by obtaining support from a dedicated organizational support group for implementation [58], providing lower-cost intervention options (eg, email based) [58], using reliable data storage methods [58], and demonstrating cost-effectiveness of the proposed intervention [64].

**Table 7:** Examples of barriers and facilitators organized under the inner setting Consolidated Framework for Implementation Research domain (N=217).

Relevant associated construct—proportion of barriers and facilitators; n (%) and brief description	Example of identified barriers	Example of identified facilitators
Structural characteristics; 4 (1.8%); the social architecture, age, maturity, and size of an organization	Personnel shortage, turnover, and organizational restructuring hindered the use of the strategy considerably [49]	Changes in the organizations should be considered (in light of resulting delays and communication problems) when planning intervention studies [42]
Networks and communications; 4 (1.8%); the nature and quality of webs of social networks and the nature and quality of formal and informal communications within an organization	Restrictive internet security settings was a barrier for accessing the intervention [42]	Conduct onsite testing before implementation [42]
Implementation climate; 17 (7.8); the absorptive capacity for change, shared receptivity of involved individuals to an intervention, and the extent to which use of that intervention will be rewarded, supported, and expected within their organization	Alignment with other stakeholders was absent and resulted in poor adherence to the recommended roles and tasks [62]	Embedding the intervention in a well-established wellness program to benefit from existing infrastructure to promote the intervention; users benefiting from incentive programs [58]
Tension for change; 1 (0.5); the degree to which stakeholders perceive the current situation as intolerable or needing change	Some stakeholders may be reluctant to implement new technology as it might threaten their ability to keep their job [39]	—^a^
Compatibility; 21 (9.7); the degree of tangible fit between meaning and values attached to the intervention by involved individuals; how those align with individuals’ own norms, values, and perceived risks and needs; and how the intervention fits with existing workflows and systems	It was not possible for employees to contact their occupational physician themselves by telephone outside their regular consultations. This could have caused difficulty when an employee struggled with a module in Return@Work and wanted to ask the occupational physician for advice [54]	Alignment to relevant stakeholders is also important and can be attained by offering ongoing support to leaders at all organizational levels during an implementation [62]
Organizational incentives and rewards; 2 (0.9); extrinsic incentives such as goal-sharing awards, performance reviews, promotions, and raises in salary and less tangible incentives such as increased stature or respect	Complimentary gifts (eg, measuring tapes to be used by users with diabetes) with logos and information stimulate discussions and act as reminders [64]	—
Readiness for implementation; 6 (2.8); tangible and immediate indicators of organizational commitment to its decision to implement an intervention	Ensuring fidelity as coaches could not provide good feedback without supervision [63]	Consult review boards and consider these issues early in the data planning process [58]
Leadership engagement; 7 (3.2); commitment, involvement, and accountability of leaders and managers with the implementation	Senior management was not engaged and too much responsibility for implementation was given to the team members who did not prioritize these activities [49]	Adherence is better when managers are active and engaged [64]
Available resources; 25 (11.5); the level of resources dedicated for implementation and ongoing operations including money, training, education, physical space, and time	The intervention required all participants to allocate the same time slot and competed with other time commitments [42]	Supporting statement from the employers which will suggest to all employees who participate in the study that they will have 1 hour per week over the 8-week period to complete the program [59]
Access to knowledge and information; 2 (0.9); ease of access to digestible information and knowledge about the intervention and how to incorporate it into work tasks	Email messages from the decision aid supported the occupational physicians when guiding employees. The email gave them sufficient information and the layout was visually attractive [54]	

^a^No facilitator reported.

#### Characteristics of Individuals

Barriers were related to either the employer or the individual (Table 8). Employer-related barriers included the perception of low organizational commitment to addressing issues targeted by the proposed intervention [49], perceived stigma associated with intervention adoption [57], and a lack of privacy (eg, sharing information disclosed within the intervention with employers) [60]. Individual-related barriers included a general lack of motivation and interest in using the intervention [40,53], no opportunities to interact with others during the intervention [57], poor consistency in using the intervention as directed [60], poor digital skills [8,41,48,49], difficulty relating to content [60], low work ability [47], and reduction in engagement and adoption due to symptoms associated with medical conditions [52,53]. Proposed facilitators include willingness to seek mental health support [50], prior experience using an eHealth intervention and interventions that are freely accessible [47], low technical skill requirement (eg, no authentication) [41], and content that is available in multiple media formats (eg, printed versions) [41,57].

**Table 8:** Examples of barriers and facilitators organized under the characteristics of individuals Consolidated Framework for Implementation Research domain (N=217).

Relevant associated construct—proportion of barriers and facilitators; n (%) and brief description	Example of identified barriers	Example of identified facilitators
Knowledge and beliefs about the intervention; 7 (3.2); individuals’ attitudes toward and the value placed on the intervention as well as familiarity with facts, truths, and principles related to the intervention	Skepticism toward the independence of the project from the organization [36]	Maintaining confidentiality between employee and employer [59]
Self-efficacy; 12 (5.5); individual belief in their own capabilities to execute courses of action to achieve implementation goals	Lack of computer skills in team members [49]	The package developed in a free-to-access and simple format that does not require logging in to a system or any specific technical expertise [41]
Other personal attributes; 6 (2.8); a broad construct to include other personal traits such as tolerance of ambiguity, intellectual ability, motivation, values, competence, capacity, and learning style	Barriers reported by participants at high risk for a major depressive episode included perceived stigma, lack of interaction with others that is characteristic of eMental health, lack of time, and lack of knowledge [57]	Willingness to seek professional mental health services [50]

#### Summary of Facilitation Measures

The identified facilitation measures were further synthesized and organized by the associated CFIR construct (Table 9).

**Table 9:** Summary of potential facilitation measures organized by associated Consolidated Framework for Implementation Research (CFIR) construct.

Associated CFIR construct	Facilitation measure
Evidence strength and quality	Strategies must provide evidence of effectiveness regarding the proposed or similar interventions in similar contexts featuring a representative sample of employees and a control group, where feasible, using valid and reliable measures.
Relative advantage	Strategies must be perceived to provide an advantage over the implementation of an alternative or no solution.
Adaptability	Strategies must allow flexibility on intervention completion times, the pace of progression, access options, and the format of provided materials.
Design quality and packaging	Strategies must ensure that the design of the intervention is based on an explicit understanding of users, their tasks, and environments and provides guidance (eg, reminders, knowledge base, progress tracking, and feedback); considers opportunities to integrate intervention features with organizational processes; creates personalized, informative, and nonstigmatizing content that encourages user participation; provides user adaptable content and tasks (ie, increased user control); allows access via additional modalities (eg, ability to print content) and formats (eg, video and audio); includes formative and summative usability testing and accessibility evaluations; highlights a strict approach to privacy and data security; and considers a multichannel recruitment strategy.
External policy and incentives	Strategies must identify and comply with applicable privacy legislation and policy regulations.
Structural characteristics	Strategies must consider the capacity of stakeholders to complete assigned tasks and account for turnover and other restructuring activities.
Networks and communications	Strategies must involve all stakeholders, include onsite testing of required technology, and establish clear communication procedures at the planning stage.
Implementation climate	Strategies must be cohesive and compatible with the organization’s culture (eg, high turnover and highly active working environment), ensure that interventions can be used in distraction-free environments (ie, free from excessive noise), account for prior negative experiences with similar interventions, secure support from senior management for strategy implementation, and leverage existing programs by embedding interventions into them.
Tension for change	Strategies must consider the impact of implementation on-the-job security of stakeholders and how that affects their perception of proposed changes.
Compatibility	Strategies must adequately reflect the implementation needs of the organization and its existing processes and policies; be aligned with stakeholders at different organizational levels; provide adequate separation between work and working with the intervention; and avoid stigmatization, especially of employees with mental health conditions.
Organizational incentives and rewards	Strategies should offer incentives for using the intervention and consider incorporating gamification components to offer these incentives.
Readiness for implementation	Strategies must ensure that stakeholders are involved in strategy development, aware of the strategy and their role in it, equipped with the necessary tools and access, and adequately trained to implement the strategy.
Leadership engagement	Strategies must secure support from all stakeholders, especially an active and engaged senior management who strongly sanctions and advocates for the intervention.
Available resources	Strategies must provide organizational support for implementation, intervention support for users, dedicated time and private spaces for completing interventions in the workplace, less time-intensive interventions, alternative options to live-participation activities (eg, live webinar recording), low-cost technology-based options (eg, email) for interventions, reliable cloud data storage, access from varying device types, and implementation cost estimates with demonstrated cost-effectiveness.
Access to knowledge and information	Strategies must provide information that sets realistic expectations about the intervention and how to implement it.
Knowledge and beliefs about the intervention	Strategies must clearly articulate the role of the organization in the development of the intervention and address privacy and stigmatization concerns associated with using mental health interventions.
Self-efficacy	Strategies must accommodate users whose performance is affected by symptoms (eg, lack of motivation) associated with their health conditions (eg, depression) and a lack of confidence using technology.
Individual identification with organization	Strategies must consider users’ perception of and level of commitment to the organization.
Other personal attributes	Strategies must address a lack of motivation (eg, due to symptoms associated with health conditions) to adopt and consistently use interventions and to seek help.

## Discussion

### Principal Findings and Comparison With Prior Work

The 31 included publications revealed 98 implementation strategies used when implementing OeMH interventions, 114 barriers, and 131 facilitators. The findings support observations [12,13] that the reporting of implementation strategies used for eHealth interventions is largely incomplete, nonsystematic, and unstructured. Nonetheless, the findings provide valuable insights into what is known and where knowledge gaps lie in the area.

### Implementation Strategies

The OeMH knowledge base does not provide definitive answers regarding the implementation strategies to adopt and when and how it is most effective and efficient to adopt them. For example, the efficacy and cost-effectiveness of using innovative methods such as web-based targeted advertising compared with traditional methods (eg, posters) to increase reach is unclear [66,67], despite the former’s success in being more time-efficient [67] and effective at recruiting hard-to-reach populations [67,68]. Those responsible for implementation must use their judgment about which of the provided strategies would be most appropriate for their circumstances. These findings support the notion that the implementation of eHealth technology (eg, eMental health [eMH] interventions) is often narrowly seen as a postdevelopment activity rather than being a crucial part of the development process [69]. Nonetheless, this could be partly a consequence of many included studies not specifically or comprehensively investigating implementation and therefore not reporting other details regarding implementation. Alternatively, publication restrictions [70] (eg, strict word limits) and the multidisciplinary nature of digital health research [71] may prioritize other study information over details regarding implementation when reporting on digital health interventions.

### Barriers and Facilitators

Similar to findings related to medical devices [72], the findings here also suggest that usability [73] appears to be the main design consideration in the evaluation of OeMH interventions, with little consideration given to other critical elements of the user experience. Findings regarding the CFIR inner setting domain highlight the need for researchers to articulate potential facilitators, including those that may have failed in one implementation context, as they might work in other contexts. Existing research [74,75] addresses many of the barriers (eg, associated with symptoms associated with mental health problems and limited digital literacy skills) categorized under the CFIR characteristics of individual domains and could provide an easy opportunity to improve implementation if given more consideration during the planning phase. Factors external to the organization (eg, external policies, partners, and competition) are known to greatly hinder or support the successful implementation of technology [76-78] but have been largely undocumented or overlooked by the included publications.

### Recency of Work and Coverage of Technologies

Similar to recent eMH reviews focusing on college students [79] and user engagement [80], this review also reported an increase since 2015 in eMH intervention studies meeting broad inclusion criteria. Recent reviews [79,80] also found that the eMH interventions described in the included studies were primarily web-based despite the added benefits of mobile apps that are coded for a specific mobile operating system such as iOS and Android (eg, faster, functionality-rich, and offline access) [81]. This is perhaps because web-based interventions likely cost less to develop and could be accessed via more devices if they were developed in a responsive way [81]. Emerging technologies, including AI, were considered in our search strategy, but were not used by the OeMH interventions described in the included studies. Nonetheless, this knowledge area is expected to increasingly feature the use of emerging technologies in the near future as the focus extends beyond nascent explorations of their applications for mental health and investigates the optimization of their implementation as well [82].

### Implications and Recommendations for Practice and Future Research

Based on the findings of the scoping review, four practical recommendations could be considered to avoid and mitigate the identified barriers and improve the implementation of OeMH interventions:

1. Strategies must demonstrate a relative advantage over alternative solutions and promote flexibility in the delivery of interventions based on an explicit understanding of users, their tasks, and environments.
2. Strategies must promote the active engagement of organizational leadership, assess organizational readiness, and ensure compatibility with the organization’s technological infrastructure and culture, in addition to providing desirable incentives and the necessary resources (eg, time and information about the intervention) for users to use the intervention as directed.
3. Strategies must ensure transparency regarding the intervention and implications of use and help users build confidence in their ability to benefit from the intervention.
4. Strategies must identify and ensure that interventions comply with applicable privacy legislation and policy regulations.

Future IR should continue with the broad aim of understanding what, why, and how OeMH interventions work under real-world conditions, and how to improve their implementation. The findings do not support the prioritization of any one aim over others. However, findings show that IR principles [83] such as the importance of context (eg, industry, size, and policies) and the people using the research need more attention for OeMH interventions. For example, surprisingly few findings were relevant to CFIR contextual domains (eg, outer setting), which speak to governmental regulations similar to COVID-19–related policies that have a strong influence on working arrangements. In addition, the general lack of detailed, systematic, and standardized reporting on proposed digital health interventions (eg, CONSORT-EHEALTH [Consolidated Standards of Reporting Trials of Electronic and Mobile Health Applications and Online Telehealth]—Expanded CONSORT figure) [32,84-86] and the implementation strategies used to achieve these outcomes (eg, Standards for Reporting Implementation Studies) [19,70] need to be remedied for IR to be properly used in this area. Reporting could benefit from subscribing to technology-centric frameworks (eg, the mobile health evidence reporting and assessment checklist [71] and the integrated technology implementation model [76-78]) that are more comprehensive in capturing key technology implementation factors (eg, accreditation, regulation, technology vendors, individual adoption factors, and interfacing systems). This should allow future studies to replicate and develop theories based on assessments of the implementation strategies used. In addition, any encountered or anticipated barriers and corresponding remedies that might be useful in avoiding these barriers or reducing their negative impact on implementation should also be reported. The development of an OeMH implementation checklist that includes comprehensive reporting guidelines and other prompts to ensure consistency and completeness when implementing these interventions would be beneficial. Future IR should also focus on investigating a wider range of common implementation outcomes (eg, cost-effectiveness and sustainability) [87] facilitated by implementation strategies for OeMH interventions that also target more common mental health problems in the workplace (eg, anxiety, substance use, and addiction). Issues regarding lack of digital access and digital inequity are an ongoing challenge [88], although not prominently featured in the results, and should be considered to avoid OeMH interventions contributing to any disparities. This study should also investigate how implementation strategies for OeMH interventions could benefit from emerging technologies. For instance, AI can use usage data to complement existing methods to better identify people who are at a high risk of mental health problems, support health decision-making, and offer resources that meet users’ individual health needs [89]. This could have a profound positive impact on implementation through improvements in the effectiveness and maintenance of interventions.

### Limitations

Search results were limited to publications in English, and a publication date restriction was imposed from 2010 onwards; however, given the broad search strategy, it is not anticipated that many, if any, potentially eligible publications were missed as a result. The term *eMental* was coined in 2002 [90], merely 8 years before this review’s year restriction, and a recent review of 50 publications about OeMH interventions [6] included 11 publications that were published before 2010 and none were eligible for inclusion in this study. In addition, despite our exhaustive search strategy, 6 publications from 2010 to 2015 compared with 25 from the subsequent 5-year period were eventually included. Incomplete reporting also made it challenging to detail strategies (eg, their effectiveness), barriers, facilitators, and contextual data (eg, industry, organizational size, and employee level) from the included publications and to synthesize these data later. Nonetheless, all researchers involved in data extraction completed the training specifically for this review, followed the same thorough approach, and the extracted data were reviewed at least once by a second researcher. Interrater reliability was not calculated, and reasons for disagreement in screening decisions were not reported, which might have affected the reproducibility of this study [91]. However, this does not compromise the consistency and accuracy of the screening. Moreover, two 2-hour workshops were conducted with training sessions, and reconciliation meetings were consequently held when there were inconsistencies in screening decisions.

Although multiple implementation strategies can legitimately contribute to multiple RE-AIM domains, adopting a framework with more specificity could potentially be useful for the identification of more targeted strategies. Common implementation models (eg, RE-AIM and CFIR) predate the current development of eHealth, and concerns about their inability to fully capture the complexities of eHealth implementation have been raised [69] and persist [92] despite some recent updates [32,86] and clarifications [33]. Nonetheless, these generic frameworks are useful for guiding data extraction and as tools for making valuable comparisons with other types of interventions. Similar to other scoping reviews, this review reports on the nature and features of the literature on the topic of focus and does not attempt to present a view regarding the appropriateness of the used methods and the strength or quality of evidence. Similarly, the provision of more detailed recommendations would have been premature and potentially misleading, as this was unsupported by the data collected. Further research is needed to determine valid facilitators and how they should be used in the process of OeMH development and delivery on a case-by-case basis while considering contextual factors such as industry, organizational size, employee level, and internal and external policies. Nevertheless, these recommendations could still be particularly relevant for OeMH interventions in comparison with similar interventions in different contexts. Consequently, readers should be mindful that the review cannot determine whether the included studies provide robust or generalizable findings.

### Conclusions

This scoping review represents one of the first steps in a research agenda aimed at improving the implementation of OeMH interventions by systematically selecting, shaping, evaluating, and reporting implementation strategies. It has identified 98 implementation strategies, 114 barriers, and 131 facilitation measures related to the implementation of these interventions. A synthesis of these findings offers 19 recommendations that provide initial guidance on how to improve the implementation of OeMH interventions. This scoping review also highlighted the need to combine common implementation models (eg, RE-AIM and CFIR) with more technology-centric frameworks (eg, integrated technology implementation model and the mobile health evidence reporting and assessment checklist) to fully capture the complexities of eHealth implementation. Despite yielding less detailed insight than hoped, owing to incomplete reporting and the adoption of incomprehensive frameworks by the included publications, this scoping review’s findings can still be critically leveraged by discerning decision-makers to improve the reach, effectiveness, adoption, implementation, and maintenance of OeMH interventions.

## Appendix

Multimedia Appendix 1 Search concepts and terms and MEDLINE search strategy.

Multimedia Appendix 2 Identified examples of implementation strategies organized by relevant reach, effectiveness, adoption, implementation, and maintenance domains.

Multimedia Appendix 3 Identified barriers and facilitators organized by relevant Consolidated Framework for Implementation Research domain and associated construct.

## Abbreviations

AI: artificial intelligence
CFIR: Consolidated Framework for Implementation Research
CONSORT-EHEALTH: Consolidated Standards of Reporting Trials of Electronic and Mobile Health Applications and Online Telehealth
eMH: eMental health
EMPOWER: European Platform to Promote Well-being and Health in the Workplace
IR: implementation research
OeMH: Occupational eMental health
PRISMA-ScR: Preferred Reporting Items for Systematic Reviews and Meta-Analyses extension for Scoping Reviews
RE-AIM: reach, effectiveness, adoption, implementation, and maintenance

## References

[1] (2017). Depression and other common mental disorders: global health estimates. World Health Organization.

[2] Young KP, Kolcz DL, O'Sullivan DM, Ferrand J, Fried J, Robinson K (2021). Health care workers' mental health and quality of life during COVID-19: results from a mid-pandemic, national survey. Psychiatr Serv.

[3] Cahill J, Cullen P, Anwer S, Wilson S, Gaynor K (2021). Pilot Work Related Stress (WRS), effects on wellbeing and mental health, and coping methods. Int J Aerospace Psychol.

[4] Sampson H, Ellis N (2020). Stepping up: the need for proactive employer investment in safeguarding seafarers’ mental health and wellbeing. Maritime Policy Manag.

[5] Hamouche S (2020). COVID-19 and employees’ mental health: stressors, moderators and agenda for organizational actions. Emerald Open Res.

[6] Phillips EA, Gordeev VS, Schreyögg J (2019). Effectiveness of occupational e-mental health interventions: a systematic review and meta-analysis of randomized controlled trials. Scand J Work Environ Health.

[7] Riper H, Andersson G, Christensen H, Cuijpers P, Lange A, Eysenbach G (2010). Theme issue on e-mental health: a growing field in internet research. J Med Internet Res.

[8] Lehr D, Geraedts A, Perrson Asplund R, Khadjesari Z, Heber E, de Bloom J, Ebert DD, Angerer P, Funk B, Wiencke M, Cacace M, Fischer S (2016). Occupational e-mental health: current approaches and promising perspectives for promoting mental health in workers. Healthy at Work: Interdisciplinary Perspectives.

[9] Ha SW, Kim J (2020). Designing a scalable, accessible, and effective mobile app based solution for common mental health problems. Int J Human Comput Interact.

[10] Rodriguez-Villa E, Naslund J, Keshavan M, Patel V, Torous J (2020). Making mental health more accessible in light of COVID-19: scalable digital health with digital navigators in low and middle-income countries. Asian J Psychiatr.

[11] Purtova N, Kosta E, Koops BJ, Fricker SA, Thümmler C, Gavras A (2014). Laws and regulations for digital health. Requirements Engineering for Digital Health.

[12] Proctor EK, Powell BJ, McMillen JC (2013). Implementation strategies: recommendations for specifying and reporting. Implement Sci.

[13] Powell BJ, McMillen JC, Proctor EK, Carpenter CR, Griffey RT, Bunger AC, Glass JE, York JL (2012). A compilation of strategies for implementing clinical innovations in health and mental health. Med Care Res Rev.

[14] Connolly SL, Hogan TP, Shimada SL, Miller CJ (2020). Leveraging implementation science to understand factors influencing sustained use of mental health apps: a narrative review. J Technol Behav Sci (forthcoming).

[15] Graham AK, Lattie EG, Powell BJ, Lyon AR, Smith JD, Schueller SM, Stadnick NA, Brown CH, Mohr DC (2020). Implementation strategies for digital mental health interventions in health care settings. Am Psychol.

[16] Dryden-Palmer KD, Parshuram CS, Berta WB (2020). Context, complexity and process in the implementation of evidence-based innovation: a realist informed review. BMC Health Serv Res.

[17] Damschroder LJ, Aron DC, Keith RE, Kirsh SR, Alexander JA, Lowery JC (2009). Fostering implementation of health services research findings into practice: a consolidated framework for advancing implementation science. Implement Sci.

[18] Varsi C, Solberg Nes L, Kristjansdottir OB, Kelders SM, Stenberg U, Zangi HA, Børøsund E, Weiss KE, Stubhaug A, Asbjørnsen RA, Westeng M, Ødegaard M, Eide H (2019). Implementation strategies to enhance the implementation of eHealth programs for patients with chronic illnesses: realist systematic review. J Med Internet Res.

[19] Pinnock H, Barwick M, Carpenter CR, Eldridge S, Grandes G, Griffiths CJ, Rycroft-Malone J, Meissner P, Murray E, Patel A, Sheikh A, Taylor SJ, StaRI Group (2017). Standards for Reporting Implementation Studies (StaRI): explanation and elaboration document. BMJ Open.

[20] (2021). EMPOWER: The European platform to promote wellbeing and health in the workplace. EMPOWER Consortium.

[21] Colquhoun HL, Levac D, O'Brien KK, Straus S, Tricco AC, Perrier L, Kastner M, Moher D (2014). Scoping reviews: time for clarity in definition, methods, and reporting. J Clin Epidemiol.

[22] Levac D, Colquhoun H, O'Brien KK (2010). Scoping studies: advancing the methodology. Implement Sci.

[23] Arksey H, O'Malley L (2005). Scoping studies: towards a methodological framework. Int J Soc Res Methodol.

[24] Khalil H, Peters M, Godfrey CM, McInerney P, Soares CB, Parker D (2016). An evidence-based approach to scoping reviews. Worldviews Evid Based Nurs.

[25] Westphaln KK, Regoeczi W, Masotya M, Vazquez-Westphaln B, Lounsbury K, McDavid L, Lee H, Johnson J, Ronis SD (2021). From Arksey and O'Malley and beyond: customizations to enhance a team-based, mixed approach to scoping review methodology. MethodsX.

[26] Tricco AC, Lillie E, Zarin W, O'Brien KK, Colquhoun H, Levac D, Moher D, Peters MD, Horsley T, Weeks L, Hempel S, Akl EA, Chang C, McGowan J, Stewart L, Hartling L, Aldcroft A, Wilson MG, Garritty C, Lewin S, Godfrey CM, Macdonald MT, Langlois EV, Soares-Weiser K, Moriarty J, Clifford T, Tunçalp Ö, Straus SE (2018). PRISMA extension for scoping reviews (PRISMA-ScR): checklist and explanation. Ann Intern Med.

[27] (2021). Global strategy on digital health 2020-2025. World Health Organization.

[28] Eysenbach G (2001). What is e-health?. J Med Internet Res.

[29] Ouzzani M, Hammady H, Fedorowicz Z, Elmagarmid A (2016). Rayyan-a web and mobile app for systematic reviews. Syst Rev.

[30] Denison HJ, Dodds RM, Ntani G, Cooper R, Cooper C, Sayer AA, Baird J (2013). How to get started with a systematic review in epidemiology: an introductory guide for early career researchers. Arch Public Health.

[31] Glasgow RE, Vogt TM, Boles SM (1999). Evaluating the public health impact of health promotion interventions: the RE-AIM framework. Am J Public Health.

[32] Glasgow RE, Harden SM, Gaglio B, Rabin B, Smith ML, Porter GC, Ory MG, Estabrooks PA (2019). RE-AIM planning and evaluation framework: adapting to new science and practice with a 20-year review. Front Public Health.

[33] Holtrop JS, Estabrooks PA, Gaglio B, Harden SM, Kessler RS, King DK, Kwan BM, Ory MG, Rabin BA, Shelton RC, Glasgow RE (2021). Understanding and applying the RE-AIM framework: clarifications and resources. J Clin Transl Sci.

[34] Powell BJ, Waltz TJ, Chinman MJ, Damschroder LJ, Smith JL, Matthieu MM, Proctor EK, Kirchner JE (2015). A refined compilation of implementation strategies: results from the Expert Recommendations for Implementing Change (ERIC) project. Implement Sci.

[35] Strifler L, Cardoso R, McGowan J, Cogo E, Nincic V, Khan PA, Scott A, Ghassemi M, MacDonald H, Lai Y, Treister V, Tricco AC, Straus SE (2018). Scoping review identifies significant number of knowledge translation theories, models, and frameworks with limited use. J Clin Epidemiol.

[36] Ravalier JM, Wainwright E, Smyth N, Clabburn O, Wegrzynek P, Loon M (2020). Co-creating and evaluating an app-based well-being intervention: the HOW (healthier outcomes at work) social work project. Int J Environ Res Public Health.

[37] Liem A, Garabiles MR, Pakingan KA, Chen W, Lam AI, Burchert S, Hall BJ (2020). A digital mental health intervention to reduce depressive symptoms among overseas Filipino workers: protocol for a pilot hybrid type 1 effectiveness-implementation randomized controlled trial. Implement Sci Commun.

[38] Freund J, Titzler I, Thielecke J, Braun L, Baumeister H, Berking M, Ebert DD (2020). Implementing Internet- and tele-based interventions to prevent mental health disorders in farmers, foresters and gardeners (ImplementIT): study protocol for the multi-level evaluation of a nationwide project. BMC Psychiatry.

[39] Engdahl P, Svedberg P, Lexén A, Bejerholm U (2020). Role of a digital return-to-work solution for individuals with common mental disorders: qualitative study of the perspectives of three stakeholder groups. JMIR Form Res.

[40] Collins DA, Harvey SB, Lavender I, Glozier N, Christensen H, Deady M (2020). A pilot evaluation of a smartphone application for workplace depression. Int J Environ Res Public Health.

[41] Blake H, Bermingham F, Johnson G, Tabner A (2020). Mitigating the psychological impact of COVID-19 on healthcare workers: a digital learning package. Int J Environ Res Public Health.

[42] Wan Mohd Yunus WM, Musiat P, Brown JS (2019). Evaluating the feasibility of an innovative self-confidence webinar intervention for depression in the workplace: a proof-of-concept study. JMIR Ment Health.

[43] Kerr DC, Ornelas IJ, Lilly MM, Calhoun R, Meischke H (2019). Participant engagement in and perspectives on a web-based mindfulness intervention for 9-1-1 telecommunicators: multimethod study. J Med Internet Res.

[44] Davey S, Gordon S, Tester R (2019). Addressing police discrimination regarding mental distress using a service user-led and interpersonal contact/education based ‘e-Learning’. Police Pract Res.

[45] Villaume K, Tafvelin S, Hasson D (2018). Health-relevant personality traits in relation to adherence to a web-based occupational health promotion and stress management intervention. Int J Workplace Health Manag.

[46] Rush KE (2018). Paving the path to mindfulness: implementation of a program to reduce stress and burnout in inpatient psychiatric nurses. University of North Carolina Digital Repository.

[47] Hennemann S, Witthöft M, Bethge M, Spanier K, Beutel ME, Zwerenz R (2018). Acceptance and barriers to access of occupational e-mental health: cross-sectional findings from a health-risk population of employees. Int Arch Occup Environ Health.

[48] Havermans BM, Boot CR, Brouwers EP, Houtman IL, Anema JR, van der Beek AJ (2018). Process evaluation of a digital platform-based implementation strategy aimed at work stress prevention in a health care organization. J Occup Environ Med.

[49] Havermans BM, Boot CR, Brouwers EP, Houtman IL, Heerkens YF, Zijlstra-Vlasveld MC, Twisk JW, Anema JR, van der Beek AJ (2018). Effectiveness of a digital platform-based implementation strategy to prevent work stress in a healthcare organization: a 12-month follow-up controlled trial. Scand J Work Environ Health.

[50] Hall BJ, Shi W, Garabiles MR, Chan EW (2018). Correlates of expected eMental Health intervention uptake among Filipino domestic workers in China. Glob Ment Health (Camb).

[51] Frykman M, Lundmark R, von Thiele Schwarz U, Villaume K, Hasson H (2018). Line managers’ influence on employee usage of a web-based system for occupational health management. Int J Workplace Health Manag.

[52] Deady M, Johnston D, Milne D, Glozier N, Peters D, Calvo R, Harvey S (2018). Preliminary effectiveness of a smartphone app to reduce depressive symptoms in the workplace: feasibility and acceptability study. JMIR Mhealth Uhealth.

[53] Carolan S, de Visser RO (2018). Employees' perspectives on the facilitators and barriers to engaging with digital mental health interventions in the workplace: qualitative study. JMIR Ment Health.

[54] Volker D, Zijlstra-Vlasveld MC, Brouwers EP, van der Feltz-Cornelis DF (2017). Process evaluation of a blended Web-based intervention on return to work for sick-listed employees with common mental health problems in the occupational health setting. J Occup Rehabil.

[55] Carolan S, Harris PR, Greenwood K, Cavanagh K (2017). Increasing engagement with an occupational digital stress management program through the use of an online facilitated discussion group: results of a pilot randomised controlled trial. Internet Interv.

[56] Zarski AC, Lehr D, Berking M, Riper H, Cuijpers P, Ebert DD (2016). Adherence to Internet-based mobile-supported stress management: a pooled analysis of individual participant data from three randomized controlled trials. J Med Internet Res.

[57] Wang J, Lam RW, Ho K, Attridge M, Lashewicz BM, Patten SB, Marchand A, Aiken A, Schmitz N, Gundu S, Rewari N, Hodgins D, Bulloch A, Merali Z (2016). Preferred features of e-mental health programs for prevention of major depression in male workers: results from a Canadian national survey. J Med Internet Res.

[58] Smith KC, Wallace DP (2016). Improving the sleep of children’s hospital employees through an email-based sleep wellness program. Clin Pract Pediatric Psychol.

[59] Carolan S, Harris PR, Greenwood K, Cavanagh K (2016). Increasing engagement with, and effectiveness of, an online CBT-based stress management intervention for employees through the use of an online facilitated bulletin board: study protocol for a pilot randomised controlled trial. Trials.

[60] Possemato K, Acosta MC, Fuentes J, Lantinga LJ, Marsch LA, Maisto SA, Grabinski M, Rosenblum A (2015). A Web-based self-management program for recent combat veterans with PTSD and substance misuse: program development and veteran feedback. Cogn Behav Pract.

[61] Schneider J, Sarrami Foroushani P, Grime P, Thornicroft G (2014). Acceptability of online self-help to people with depression: users' views of MoodGYM versus informational websites. J Med Internet Res.

[62] Hasson H, Villaume K, von Thiele Schwarz U, Palm K (2014). Managing implementation: roles of line managers, senior managers, and human resource professionals in an occupational health intervention. J Occup Environ Med.

[63] Geraedts AS, Kleiboer AM, Wiezer NM, Cuijpers P, van Mechelen W, Anema JR (2014). Feasibility of a worker-directed Web-based intervention for employees with depressive symptoms. Internet Interv.

[64] Hasson D, Villaume K, Bauer GF, Jenny GJ (2013). An automated and systematic Web-based intervention for stress management and organizational health promotion. Salutogenic Organizations and Change.

[65] Hasson H, Brown C, Hasson D (2010). Factors associated with high use of a workplace web-based stress management program in a randomized controlled intervention study. Health Educ Res.

[66] Reagan L, Nowlin SY, Birdsall SB, Gabbay J, Vorderstrasse A, Johnson C, D'Eramo Melkus G (2019). Integrative review of recruitment of research participants through Facebook. Nurs Res.

[67] Kayrouz R, Dear BF, Karin E, Titov N (2016). Facebook as an effective recruitment strategy for mental health research of hard to reach populations. Internet Interv.

[68] Whitaker C, Stevelink S, Fear N (2017). The use of Facebook in recruiting participants for health research purposes: a systematic review. J Med Internet Res.

[69] van Gemert-Pijnen L, Kelders SM, Kip H, Sanderman R (2018). eHealth Research, Theory and Development: A Multidisciplinary Approach.

[70] Pinnock H, Barwick M, Carpenter CR, Eldridge S, Grandes G, Griffiths CJ, Rycroft-Malone J, Meissner P, Murray E, Patel A, Sheikh A, Taylor SJ, StaRI Group (2017). Standards for Reporting Implementation Studies (StaRI) statement. BMJ.

[71] Agarwal S, LeFevre AE, Lee J, L'Engle K, Mehl G, Sinha C, Labrique A, WHO mHealth Technical Evidence Review Group (2016). Guidelines for reporting of health interventions using mobile phones: mobile health (mHealth) evidence reporting and assessment (mERA) checklist. BMJ.

[72] Bitkina OV, Kim HK, Park J (2020). Usability and user experience of medical devices: an overview of the current state, analysis methodologies, and future challenges. Int J Ind Ergon.

[73] (2018). Ergonomics of human-system interaction — Part 304: user performance test methods for electronic visual displays - ISO 9241-304:2008. International Organization for Standardization.

[74] Bernard R, Sabariego C, Cieza A (2019). Difficulties encountered by people with depression and anxiety on the Web: qualitative study and Web-based expert survey. J Med Internet Res.

[75] Bernard R, Sabariego C, Cieza A (2016). Barriers and facilitation measures related to people with mental disorders when using the web: a systematic review. J Med Internet Res.

[76] Schoville RR (2017). Discovery of implementation factors that lead to technology adoption in long-term care. J Gerontol Nurs.

[77] Schoville R, Titler MG (2020). Integrated technology implementation model: examination and enhancements. Comput Inform Nurs.

[78] Schoville RR, Titler MG (2015). Guiding healthcare technology implementation: a new integrated technology implementation model. Comput Inform Nurs.

[79] Lattie EG, Adkins EC, Winquist N, Stiles-Shields C, Wafford QE, Graham AK (2019). Digital mental health interventions for depression, anxiety, and enhancement of psychological well-being among college students: systematic review. J Med Internet Res.

[80] Borghouts J, Eikey E, Mark G, De Leon C, Schueller SM, Schneider M, Stadnick N, Zheng K, Mukamel D, Sorkin DH (2021). Barriers to and facilitators of user engagement with digital mental health interventions: systematic review. J Med Internet Res.

[81] Panhale M (2016). Beginning Hybrid Mobile Application Development.

[82] Baños RM, Herrero R, Vara MD (2022). What is the current and future status of digital mental health interventions?. Span J Psychol.

[83] Peters DH, Adam T, Alonge O, Agyepong IA, Tran N (2013). Implementation research: what it is and how to do it. BMJ.

[84] Eysenbach G, CONSORT-EHEALTH Group (2011). CONSORT-EHEALTH: improving and standardizing evaluation reports of Web-based and mobile health interventions. J Med Internet Res.

[85] Eysenbach G (2013). CONSORT-EHEALTH: implementation of a checklist for authors and editors to improve reporting of Web-based and mobile randomized controlled trials. Studies in Health Technology and Informatics.

[86] Glasgow RE, Huebschmann AG, Brownson RC (2018). Expanding the CONSORT figure: increasing transparency in reporting on external validity. Am J Prev Med.

[87] Proctor E, Silmere H, Raghavan R, Hovmand P, Aarons G, Bunger A, Griffey R, Hensley M (2011). Outcomes for implementation research: conceptual distinctions, measurement challenges, and research agenda. Adm Policy Ment Health.

[88] Crawford A, Serhal E (2020). Digital health equity and COVID-19: the innovation curve cannot reinforce the social gradient of health. J Med Internet Res.

[89] Ebert DD, Harrer M, Apolinário-Hagen J, Baumeister H (2019). Digital interventions for mental disorders: key features, efficacy, and potential for artificial intelligence applications. Adv Exp Med Biol.

[90] Christensen H, Griffiths K, Evans K (2002). E-Mental Health in Australia: Implications of the Internet and Related Technologies for Policy.

[91] Belur J, Tompson L, Thornton A, Simon M (2018). Interrater reliability in systematic review methodology: exploring variation in coder decision-making. Sociol Methods Res.

[92] Heinsch M, Wyllie J, Carlson J, Wells H, Tickner C, Kay-Lambkin F (2021). Theories informing eHealth implementation: systematic review and typology classification. J Med Internet Res.

