# Supplementary material for: Strategies for Implementing Occupational eMental Health Interventions: Scoping Review
Source: J Med Internet Res. 2022 Jun 1;24(6):e34479. doi: 10.2196/34479 (PMC9201704; doi:10.2196/34479)
Supplement: Multimedia Appendix 1 [file jmir_v24i6e34479_app1.docx]

Multimedia Appendix 1. Search concepts and terms, and MEDLINE search strategy

**Summary of search concepts and terms**

| **Concepts** | **Search Terms** |
| --- | --- |
| mental health | S1 (detailed in the MEDLINE search strategy below) |
| digital | S2 (detailed in the MEDLINE search strategy below) |
| workplace | S3 (detailed in the MEDLINE search strategy below) |
| implementation/barrier/facilitator | S4 (detailed in the MEDLINE search strategy below) |

**MEDLINE search strategy**

| S5 | S1 AND S2 AND S3 AND S4 |
| --- | --- |
| S4 | TI ("delivery of health care" OR "delivery of healthcare" OR "evidence to practice" OR "health care reform" OR "health planning" OR "knowledge to practice" OR "knowledge transfer" OR "evaluation research" OR "organisational innovation" OR "organizational innovation" OR "perceived usefulness" OR "process evaluation*" OR "program evaluation*" OR "technology transfer" OR "user experience" OR adapt OR adaptation OR adoption OR barrier* OR deterrent* OR diffusion OR disseminat* OR facilitate* OR facilitating OR facilitator* OR hindrance* OR hurdle* OR implement* OR integrate* OR obstacle* OR obstruct OR promote* OR sustain* OR translation* OR translational OR upscale OR up-scale OR uptake OR up-take OR user-experience OR utilis* OR utiliz* OR strateg* OR compliance* OR accept* OR conform* OR approv* OR adherence* OR guideline* OR guidance* OR evaluat* OR usage* OR improve* OR improva* OR problem-solving OR "problem solving" OR limitation* OR promoti* OR administer*) OR AB ("delivery of health care" OR "delivery of healthcare" OR "evidence to practice" OR "health care reform" OR "health planning" OR "knowledge to practice" OR "knowledge transfer" OR "evaluation research" OR "organisational innovation" OR "organizational innovation" OR "perceived usefulness" OR "process evaluation*" OR "program evaluation*" OR "technology transfer" OR "user experience" OR adapt OR adaptation OR adoption OR barrier* OR deterrent* OR diffusion OR disseminat* OR facilitate* OR facilitating OR facilitator* OR hindrance* OR hurdle* OR implement* OR integrate* OR obstacle* OR obstruct OR promote* OR sustain* OR translation* OR translational OR upscale OR up-scale OR uptake OR up-take OR user-experience OR utilis* OR utiliz* OR strateg* OR compliance* OR accept* OR conform* OR approv* OR adherence* OR guideline* OR guidance* OR evaluat* OR usage* OR improve* OR improva* OR problem-solving OR "problem solving" OR limitation* OR promoti* OR administer*) OR (MH implementation science) OR (MH diffusion of innovation) OR (MH communication barriers) OR (MH systems implementation) |
|  |  |
|  |  |
| S3 | TI (absenteeism OR burnout OR business* OR career OR companies OR company OR corporate OR employe* OR employment* OR enterprise OR enterprises OR job OR jobs OR labor OR labour OR occupation OR occupational OR occupations OR presenteeism OR professional* OR staff OR staffe* OR staffing OR staffs OR vocation OR vocational OR vocations OR work OR work/life OR worker OR workers OR workforce* OR work-life OR workload* OR workm* OR workplace* OR worksite* OR worksite-based OR workspace* OR workstation* OR training OR working) OR AB (absenteeism OR burnout OR business* OR career OR companies OR company OR corporate OR employe* OR employment* OR enterprise OR enterprises OR job OR jobs OR labor OR labour OR occupation OR occupational OR occupations OR presenteeism OR professional* OR staff OR staffe* OR staffing OR staffs OR vocation OR vocational OR vocations OR work OR work/life OR worker OR workers OR workforce* OR work-life OR workload* OR workm* OR workplace* OR worksite* OR worksite-based OR workspace* OR workstation* OR training OR working) OR (MH "absenteeism") OR (MH "burnout, professional") OR (MH "employment") OR (MH "employment/PF") OR (MH "job re-entry") OR (MH "occupational diseases/PC/PF") OR (MH "occupational health") OR (MH "occupational medicine/MT") OR (MH "presenteeism") OR (MH "stress, occupational") OR (MH "work environment") OR (MH "work") OR (MH "workload") |
|  |  |
|  |  |
| S2 | TI ("access to information" OR "artificial intelligence" OR "cell phone" OR "computer-based*" OR "consumer health informatics" OR "digital application*" OR "electronic communication*" OR "electronic mail" OR "electronic messag*" OR "electronic reminder*" OR "information system*" OR "machine learning" OR "medical informatics" OR "medical record system*" OR "message reminder*" OR "mobile application*" OR "mobile healthcare" OR "mobile technology" OR "monitoring device*" OR "monitoring sensor" OR "monitoring sensors" OR "multimedia" OR "neural network*" OR "remote consultation*" OR "smart device*" OR "smart glasses" OR "smart phone*" OR "social media" OR "social networking" OR "text messag*" OR "web page*" OR "world wide web" OR app OR apps OR automated OR blog* OR computer* OR computer-aided OR device* OR digital OR ehealth OR e-health OR elearning OR e-learning OR electronic OR electronics OR email* OR e-mail* OR emental OR e-mental OR facebook OR ict OR innovation* OR internet OR internet-based OR mhealth OR m-health OR mobile-phone OR net OR online OR on-line OR phone OR phone-based OR portal* OR smartphone-based OR software OR technologies OR technology OR technology-based OR telecommunication* OR teleconsultation OR tele-health OR telemedicine OR telemonitoring OR telephone OR telerehabilitation OR wearable* OR web OR web-based OR website* OR whatsapp OR wireless OR www OR tele-monitor* OR tele-rehabilitation OR tele-consultation OR tele-medicine OR telehealth OR tele-care OR telecare OR telemonitor* OR web-portal*) OR AB ("access to information" OR "artificial intelligence" OR "cell phone" OR "computer-based*" OR "consumer health informatics" OR "digital application*" OR "electronic communication*" OR "electronic mail" OR "electronic messag*" OR "electronic reminder*" OR "information system*" OR "machine learning" OR "medical informatics" OR "medical record system*" OR "message reminder*" OR "mobile application*" OR "mobile healthcare" OR "mobile technology" OR "monitoring device*" OR "monitoring sensor" OR "monitoring sensors" OR "multimedia" OR "neural network*" OR "remote consultation*" OR "smart device*" OR "smart glasses" OR "smart phone*" OR "social media" OR "social networking" OR "text messag*" OR "web page*" OR "world wide web" OR app OR apps OR automated OR blog* OR computer* OR computer-aided OR device* OR digital OR ehealth OR e-health OR elearning OR e-learning OR electronic OR electronics OR email* OR e-mail* OR emental OR e-mental OR facebook OR ict OR innovation* OR internet OR internet-based OR mhealth OR m-health OR mobile-phone OR net OR online OR on-line OR phone OR phone-based OR portal* OR smartphone-based OR software OR technologies OR technology OR technology-based OR telecommunication* OR teleconsultation OR tele-health OR telemedicine OR telemonitoring OR telephone OR telerehabilitation OR wearable* OR web OR web-based OR website* OR whatsapp OR wireless OR www OR tele-monitor* OR tele-rehabilitation OR tele-consultation OR tele-medicine OR telehealth OR tele-care OR telecare OR telemonitor* OR web-portal*) OR (MH "access to information") OR (MH "blogs") OR (MH "cellular phone") OR (MH "computer systems") OR (MH "computers and computerization") OR (MH "computers, hand-held") OR (MH "educational technology") OR (MH "information technology") OR (MH "internet access") OR (MH "internet") OR (MH "mobile applications") OR (MH "smart glasses") OR (MH "smartphone") OR (MH "social media") OR (MH "social networking") OR (MH "software") OR (MH "telecommunications") OR (MH "telemedicine/MT") OR (MH "telephone") OR (MH "wearable sensors") OR (MH "wireless communications") OR (MH computer assisted Instruction) |
|  |  |
|  |  |
| S1 | TI ("bipolar disorder" OR "cognitive behavioral therapy" OR "eating disorder*" OR "health management" OR "mental disorder*" OR "mental health" OR "mental ill health" OR "mental illness*" OR "mental practice" OR "mood disorder*" OR "personality disorder*" OR "post-traumatic" OR "psychiatric illness*" OR "psychotic disorder*" OR "quality of life" OR "well being" OR anxiety OR bipolar OR burnout OR burn-out OR depressed OR depression OR depressive OR dissociative OR intervention* OR mania OR manic OR mindfulness OR neurocognitive OR obsessive-compulsive OR panic OR phobia* OR psycholog* OR schizophrenia OR stress OR therapy OR treatment* OR wellbeing OR well-being) OR AB ("bipolar disorder" OR "cognitive behavioral therapy" OR "eating disorder*" OR "health management" OR "mental disorder*" OR "mental health" OR "mental ill health" OR "mental illness*" OR "mental practice" OR "mood disorder*" OR "personality disorder*" OR "post-traumatic" OR "psychiatric illness*" OR "psychotic disorder*" OR "quality of life" OR "well being" OR anxiety OR bipolar OR burnout OR burn-out OR depressed OR depression OR depressive OR dissociative OR intervention* OR mania OR manic OR mindfulness OR neurocognitive OR obsessive-compulsive OR panic OR phobia* OR psycholog* OR schizophrenia OR stress OR therapy OR treatment* OR wellbeing OR well-being) OR (MH "cognitive therapy/MT") OR (MH "depression/TH") OR (MH "health care delivery") OR (MH "mental disorders /TH/PC/PF") OR (MH "mental health services") OR (MH "mental health/TD/MT/UT") OR (MH "mindfulness") OR (MH "occupational health") OR (MH "psychotherapy/MT/PF/TH") OR (MH "self care") |
